# Supplementary material for: “To Be Treated as a Person and Not as a Disease Entity”—Expectations of People with Visual Impairments towards Primary Healthcare: Results of the Mixed-Method Survey in Poland
Source: Int J Environ Res Public Health. 2022 Oct 19;19(20):13519. doi: 10.3390/ijerph192013519 (PMC9602634; doi:10.3390/ijerph192013519)
Supplement: Supplementary file 1 [file ijerph-19-13519-s001.zip › ijerph-1931633-supplementary.pdf]

Supplementary Materials

Binder-Olibrowska, K.W.; Godycki-Ćwirko, M.; Wrzesińska, M.A. "To Be Treated as a Person and Not as a Disease Entity"—Expectations of People with Visual Impairments towards Primary Healthcare: Results of the Mixed-Method Survey in Poland.

Table S1: Changes made in PVQ after a pilot study among PVIs.

Questions in the previous Polish study [23]	Changes made in the current study and their rationale
Participants characteristics questions	
• age,<br>• gender,<br>• ethnicity,<br>• mother language proficiency,<br>• presence of other adults and children in a shared household,<br>• employment status/occupational position,<br>• education level,<br>• income level,<br>• self-assessment of health status,<br>• quality of life,<br>• diagnosis of disease or condition such as high blood pressure, diabetes, depression, asthma or another longstanding condition)	The questions about the respondent's and mother's place of birth and a question about how well the surveyed speak Polish were omitted.<br>We assumed that similarly to the Polish study [23], the surveyed group was highly likely to be homogeneous in this respect or, if people of diverse ethnic or linguistic backgrounds appeared, describing them in such a narrow group might reveal their anonymity.
	Pensioners were included in the item concerning the current employment situation. We assumed that in the group of PwDs this category may appear more often than in the general population.<br>Additionally, we asked if respondents were poor-sighted or blind, and whether they had additional disabilities (multiple answers could be selected from the following categories: no other disability, hearing impairment, mobility impairment, and another disability but for analyses the responses were reduced to yes and no).<br>Participants also ticked the way of moving around in public spaces, choosing one or more options out of: without any outside help or assistance, with the help of a guide/assistant, a white cane; a guide dog; using GPS, other, and independently, without external assistance or aids. Only the last one response was considered independent mobility in the further analysis.<br>Moreover, we asked if respondents use the service of an assistant of PwDs often, seldom or rarely.
PHC values questions	
the word "practice" repeated in several questions	The word "practice" (Polish "praktyka"), unintelligible to some of those included in the pilot survey, was replaced with the "clinic center" (Polish "poradnia").
"[how important is it] that you are able to bring a family member/friend to your appointment if you find it useful"	The item was expanded to include "an assistant or other people"
"[how important is it] that the doctor is aware of your social, cultural background" and "so that the doctor is not prejudiced because of your age, gender, religion, cultural background"	To these items "disability" was added.
"[how important is it] "that the doctor gives you additional information about your health problem, e.g. by giving leaflets"	An item was complemented with: "or other educational materials adapted to visual problems"
"[how important is it] „to be prepared to ask questions and take notes"	An item was complemented with: "also using tools adapted for visual impairment."

Table S2: Frequency of use of primary care physician services in the past six months.

	At all		Once		Two times		3-5 times		6 or more times		Total	
	n	%	n	%	n	%	n	%	n	%	n	%
						General						
Poor-sighted	22	14.4	15	9.8	39	25.5	52	34.0	25	16.3	153	71.2
The blind	17	27.5	9	14.5	15	24.2	12	19.4	9	14.5	62	28.8
						Poor-sighted						
Women	12	12.4	12	12.4	21	21.6	33	34.0	19	19.6	97	63.4
Men	10	17.9	3	5.4	18	32.1	19	33.9	6	10.7	56	36.6
						The blind						
Women	8	29.7	3	11.1	7	25.9	6	22.2	3	11.1	27	43.5
Men	9	25.8	6	17.1	8	22.9	6	17.1	6	17.1	35	56.5
						Disability						
Visual impairment and other disability	12	14.3	9	10.7	17	20.3	27	32.1	19	22.6	84	39.1
Only visual impairment	27	20.6	15	11.5	37	28.2	37	28.2	15	11.5	131	60.9
						Chronic disease [1]						
Yes	18	13.2	13	9.6	29	21.3	48	35.3	28	20.6	136	63.3
No	21	26.6	11	13.9	25	31.7	16	20.2	6	7.6	79	36.7

[1] $chi^2=17.013$; $p=0.002$.
